# Prediction of HER2 Status Based on Deep Learning in H&E-Stained Histopathology Images of Bladder Cancer

**DOI:** 10.3390/biomedicines12071583

**Published:** 2024-07-17

**Authors:** Panpan Jiao, Qingyuan Zheng, Rui Yang, Xinmiao Ni, Jiejun Wu, Zhiyuan Chen, Xiuheng Liu

**Affiliations:** 1 Department of Urology, Renmin Hospital of Wuhan University, Wuhan 430060, China; 2022283020231@whu.edu.cn (P.J.); zqy710890394@whu.edu.cn (Q.Z.); yang20085935@whu.edu.cn (R.Y.); drnxm1997@whu.edu.cn (X.N.); 2018305231076@whu.edu.cn (J.W.); 2 Institute of Urologic Disease, Renmin Hospital of Wuhan University, Wuhan 430060, China

**Keywords:** bladder cancer, HER2, deep learning, histopathology image

## Abstract

Epidermal growth factor receptor 2 (*HER2*) has been widely recognized as one of the targets for bladder cancer immunotherapy. The key to implementing personalized treatment for bladder cancer patients lies in achieving rapid and accurate diagnosis. To tackle this challenge, we have pioneered the application of deep learning techniques to predict *HER2* expression status from H&E-stained pathological images of bladder cancer, bypassing the need for intricate IHC staining or high-throughput sequencing methods. Our model, when subjected to rigorous testing within the cohort from the People’s Hospital of Wuhan University, which encompasses 106 cases, has exhibited commendable performance on both the validation and test datasets. Specifically, the validation set yielded an AUC of 0.92, an accuracy of 0.86, a sensitivity of 0.87, a specificity of 0.83, and an F1 score of 86.7%. The corresponding metrics for the test set were 0.88 for AUC, 0.67 for accuracy, 0.56 for sensitivity, 0.75 for specificity, and 77.8% for F1 score. Additionally, in a direct comparison with pathologists, our model demonstrated statistically superior performance, with a *p*-value less than 0.05, highlighting its potential as a powerful diagnostic tool.

## 1. Introduction

Bladder cancer is currently the seventh most prevalent cancer globally and ranks 13th in terms of cancer-related mortality. As per the statistics released by the American Cancer Society for the year 2023, it is estimated that there will be around 82,290 new cases of bladder cancer diagnosed in the United States. Consequently, bladder cancer is expected to become the fourth most common cancer impacting men’s health in the country [1]. Platinum-based chemotherapy is a first-line treatment option for locally advanced and metastatic urothelial carcinoma. After undergoing first-line therapy, it becomes necessary for patients experiencing disease progression to explore further diagnostic and treatment options.

Targeted therapy against human epidermal growth factor receptor 2 (*HER2*), also known as tyrosine kinase receptor erbB-2, is one of the objectives within the scope of second-line treatment. HER2 is a transmembrane glycoprotein with vital membrane tyrosine kinase activity, playing a critical role in controlling the growth and differentiation of epithelial cells [2]. HER2 is a crucial cancer biomarker, as its activation plays a role in promoting angiogenesis and tumor formation. Overexpression of *HER2* has been observed in various adenocarcinomas, spanning breast cancer, bladder cancer, ovarian cancer, endometrial cancer, cervical cancer, lung cancer, esophageal cancer, and gastric cancer.

Studies have shown that *HER2* plays an important role in the development and progression of urothelial carcinoma [3]. In the context of bladder urothelial carcinoma, the proportion of *HER2* overexpression (immunohistochemistry staining 2+ and 3+) ranges from 18.1% to 36% [4,5,6]. Overexpression of *HER2* is closely related to bladder cancer progression and poor prognosis [7,8]. A phase II clinical study focused on locally advanced bladder cancer and metastatic urothelial carcinoma that is *HER2*-positive (HER2 2+ and 3+) demonstrated the effectiveness of RC048-ADC treatment, with an evaluated overall response rate of 51.2%. Additionally, there were extensions observed in both median progression-free survival (PFS) and median overall survival (OS) [9]. Xu et al. reported a case of a 68-year-old elderly male diagnosed with PD-LI (–), HER2 (3+), and renal impairment. After the failure of first-line platinum-based chemotherapy, the patient underwent treatment with RC48, a *HER2*-targeting antibody–drug conjugate, in combination with pembrolizumab. Following this treatment regimen, the patient achieved a rapid partial response upon the first assessment and was found to have extended progression-free survival during follow-up [10]. In conclusion, anti-*HER2* antibody–drug conjugates (ADCs) have shown promising efficacy and safety in treating patients with *HER2* overexpressing locally advanced or metastatic urothelial carcinoma. In the decision-making process for the treatment of metastatic urothelial carcinoma and locally advanced urothelial carcinoma, *HER2*-targeted antibody–drug conjugates and immune checkpoint inhibitors have entered the scope of international guidelines such as EAU and NCCN [11,12].

It is evident that the efficacy of the aforementioned treatment approaches hinges on accurate identification of *HER2* expression status. Although high-throughput sequencing can be used to predict the *HER2* expression status in bladder cancer tissue, it is mostly employed for qualitative assessment and is costly. The staining of HER2 in the context of cancer diagnosis mainly relies on the immunohistochemistry (IHC) technique, a traditional and widely used method for quantitative evaluation. However, IHC can be resource-intensive in terms of both time and cost, and not all medical facilities may have the necessary expertise and equipment to perform it effectively. Additionally, the interpretation of HER2 IHC staining is entirely dependent on the expertise of pathologists, despite the existence of consensus guidelines for interpreting HER2 immunohistochemistry staining [13,14]. Nevertheless, this still relies on the knowledge and professional level of pathologists. Numerous studies have indicated that *HER2* expression is often low and exhibits high heterogeneity, and this heterogeneity is correlated with disease-free survival (DFS) and overall survival (OS) [15,16,17,18,19].

As is widely acknowledged, hematoxylin and eosin (H&E) staining is the fundamental and standard technique for tissue and cell staining. Pathologists can visually assess and categorize lesions by directly examining tissue slides. H&E staining is more efficient, cost-effective, and reliable when compared to immunohistochemistry (IHC) staining. However, it is important to note that, currently, pathologists cannot determine or predict *HER2* expression solely through visual observation. Additional specific tests, such as IHC, are required to accurately assess *HER2* expression levels in tissues.

However, there is a growing body of evidence demonstrating that deep learning can effectively capture features in H&E images that may not be easily identifiable by the human eye. For instance, more recent research has also utilized CNN to predict the expression of ER, progesterone receptor, and *HER2* in breast cancer from H&E-stained whole slide images (WSIs) [20,21,22], as well as to predict PD-L1 expression [23].

Deep learning is a specialized field within artificial intelligence that harnesses advanced algorithms like convolutional neural networks (CNNs) to uncover intricate patterns within vast datasets and fit data features through backpropagation. This enables the development of sophisticated deep learning models applicable in diverse areas, such as disease diagnosis, cancer target analysis, drug design and development, and survival prediction [24,25,26]. In computer vision-guided pathological research, a lot of deep learning models have demonstrated performance comparable to that of pathologists [27,28,29].

Yan et al. introduced a hierarchical deep multiple-instance learning framework that has been applied to predict the *HER2* expression status in bladder cancer tissues, as annotated by pathologists within the TCGA dataset, which comprises 123 cases. The model demonstrated impressive performance, with an area under the receiver operating characteristic curve (AUC) reaching a remarkable 0.91 [30]. Farahmand et al. used CNN to predict *HER2* status in breast cancer on WSIs and their AUC reached 0.80 in five-fold cross-validation [22]. Che et al. used deep learning to predict *HER2* status in breast cancer on WSIs and the accuracy of classification performance at patch-level on the test set was 73.49% [31]. Despite many similar studies having predicted *HER2* expression and achieving good performance in breast cancer and other tumors, the universality of the model has yet to be demonstrated [32,33,34].

However, despite these advancements, there is currently no evidence suggesting that deep learning-based analysis of H&E images can be used for quantitative prediction of *HER2* expression status in bladder cancer. We decided to deliver an economical, swift, and precise diagnostic solution that integrates seamlessly into digital pathology workflows. This innovation can mitigate the heterogeneity associated with immunohistochemical staining, paving the way for early and targeted treatment for those with *HER2*-positive status.

Hence, in this study, we developed a weakly-supervised deep learning model using a clustering-constrained-attention multiple-instance learning (CLAM) framework to predict *HER2* status from routine H&E-stained slides of bladder cancer from an RHWU cohort and attempted to identify new histopathological features. The cohort was divided into training, validation, and test sets. We used a five-fold cross-validation strategy to train the deep learning model. The model’s performance was evaluated on the validation and test sets using metrics such as sensitivity, specificity, accuracy, and area under the receiver operating characteristic curve (AUC).

The structure of this study unfolds as follows. Initially, we set the stage by providing the study’s context and reviewing the pertinent literature. Subsequently, we delineate the materials and methodologies employed. Section 3 is dedicated to presenting and elucidating the findings. In the penultimate part, we engage in a comprehensive discussion of the research. Concluding the paper, we offer insights for future inquiries.

## 2. Materials and Methods

### 2.1. Patient Cohort

**Figure 1:**
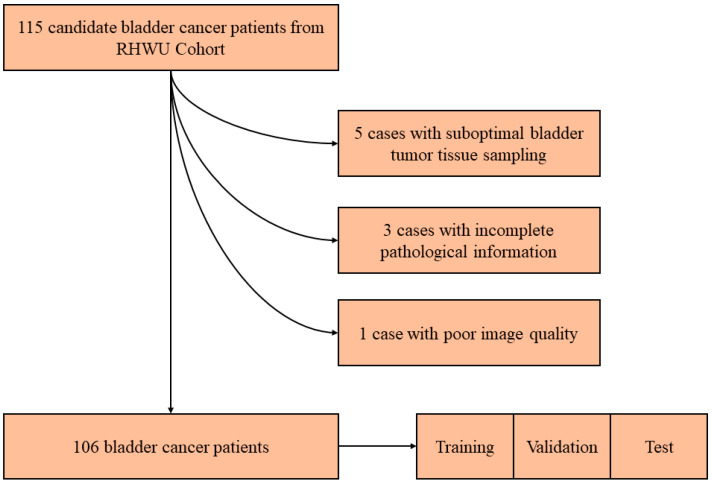
Description of pathways to recruit patients from the RHWU cohort.

We received approval from the Clinical Research Ethics Committee of RHWU for our retrospective study, adhering to the principles of the Helsinki Declaration, and obtaining informed consent from all participants (Ethical Approval Number: WDRY2022-K077). We collected H&E-stained pathology slides from a total of 115 patients who underwent bladder tumor surgery at RHWU between the years 2020 and 2023. These H&E-stained slides were meticulously prepared by skilled technicians and evaluated individually by molecular pathologists. The inclusion criteria are as follows: (a) Only cases with a definitive pathological diagnosis of bladder cancer, available pathological images or blocks, (b) known *HER2* status, (c) no history of targeted or immunotherapy, and (d) clinical data regarding age, gender, T stage, lymphovascular invasion, and histologic grade were included (Table 1). The patient recruitment pathway is shown in Figure 1.

In the preparation of H&E-stained slides, meticulous attention is paid to ensuring that the sections are intact, uniformly thin, and devoid of any knife marks, tremors, wrinkles, folds, or bubbles. There should be no excess glue, and the slides must be free from contamination. The sections should exhibit excellent transparency, with a sharp contrast between the nucleus and cytoplasm, and a balanced red-blue coloration. During the scanning process, it is imperative to adhere strictly to the scanner’s manual for proper operation. Each whole slide image (WSI) must be meticulously checked, named, and catalogued to ensure that the post-scanning WSI data is in complete alignment with the corresponding patient case, guaranteeing accuracy and consistency.

Immunohistochemistry staining was employed to determine the *HER2* status and the score was performed according to the current ASCO/College of American Pathologists guidelines for scoring HER2. An IHC staining score of 3+ or 2+ was deemed positive, while patients with scores of 0 or 1+ were classified as negative. Sample images to the dataset description can be founded in the Supplementary Materials.

### 2.2. WSI Preprocessing

We enlisted the expertise of molecular pathologists to thoroughly assess tissue pathology slides from 115 bladder cancer patients. Following the evaluation, nine cases were excluded from the RHWU cohort due to various reasons, including poor image quality (1/9), suboptimal bladder tumor tissue sampling (5/9), and incomplete pathological information (3/9). The remaining qualified tissue pathology slides were then selected for scanning.

After careful evaluation by molecular pathologists, each of the 106 tissue pathology slides from bladder cancer patients was scanned into a corresponding WSI in .svs format using a digital scanner (KF-PRO-020, KFBIO Co., Ltd., Ningbo, China), and carefully reviewed by pathologists. Subsequently, all WSIs underwent meticulous inspection before being securely stored in an external storage system for further analysis.

### 2.3. WSI Segmentation

After importing the 106 WSIs in .svs format at 20× magnification, our initial step involved segmenting the boundaries of bladder cancer tissues and identifying natural pores on the tissue pathology slide images. Subsequently, we divided the digital tissue pathology slides into patches and filtered out blank patches using color thresholding techniques.

All patches are transformed into a low-dimensional feature embedding set, which is then fed into the attention network. Feature extraction was performed by the ResNet-50 model with ImageNet pre-training weights. Thus, 1024 features could be generated for each patch through the feature extractor.

### 2.4. Model Development

**Figure 2:**
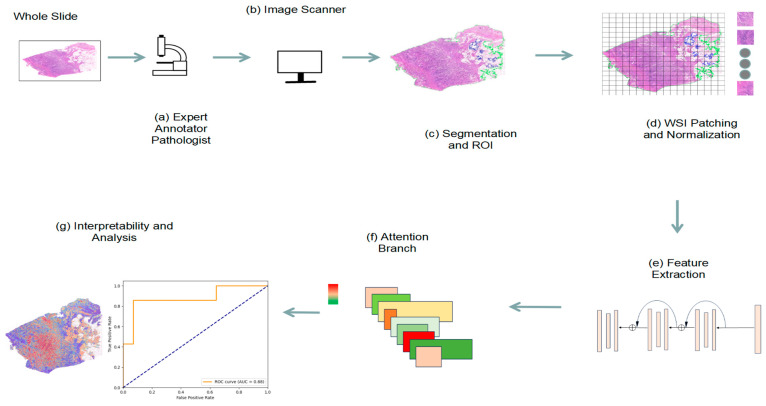
The whole process of the research workflow is shown. (**a**) Pathologists annotate the pathological slides of bladder cancer one by one; (**b**) scan the histopathological slides into digital pathological sections and transfer them to external storage; (**c**) segment the digital pathological slides and mark ROI (green line is tissue boundary, blue line is holes); (**d**) WSI patching and normalization; (**e**) feature extraction; (**f**) attention branch; (**g**) heatmap visualization and analysis.

CLAM is an advanced weakly supervised deep learning method that leverages attention learning to automatically identify subregions with significant diagnostic value [35]. By employing instance-level clustering on the identified representative regions, it effectively constrains and refines the feature space, leading to precise classification of WSIs [28]. CLAM can classify unannotated WSIs by using an attention-based pooling function. Here, we show the whole process of the whole research work (Figure 2).

To train the deep learning model, we used a five-fold cross-validation strategy for repeated validation to prevent overfitting. Five-fold cross-validation stands as a powerful and reliable method for gauging a model’s ability to generalize. It achieves this by segmenting the dataset into five equal parts, utilizing one part as the validation set in each round while the other four parts constitute the training set. This approach ensures a more consistent and dependable assessment of the model’s performance, confirming its stability and efficacy across various data subsets. The Adam optimizer with an initial learning rate of 1 × 10^−4^ and ℓ2 weight decay of 1  ×  10^−5^ was used to update the training weights and parameters. The remaining hyperparameters were set to β_1_ of 0.9 and β_2_ of 0.999. The input feature dimension, hidden layer dimension, and dropout rate were set to 1024, 256, and 0.25, respectively. The loss function was selected using a smooth top-1 SVM loss. The maximum training epoch was set to 200, and when the loss of SBLNP did not change for 20 consecutive epochs, the early stopping strategy was used to stop training and saved the best model for nest validation.

### 2.5. Model Predictions by Attention Heatmap

The attention network assigned an attention score to each patch, indicating their relative importance in the diagnosis of the entire slide. These attention scores were converted into percentile scores and scaled to between 0 and 1 (1 being the highest attendance and 0 being the lowest). A diverging color map was used to convert normalized scores to RGB colors and was displayed at the top of the respective spatial locations in the slide to visually identify and explain areas of high interest shown in red (positive evidence, high contribution to model predictions) and low interest shown in blue (low contribution to model predictions relative to other patches). Once the training was completed, the model could identify feature-rich regions and perform classification at the WSI level.

### 2.6. Statistical Analysis

In our study, we collected a total of 106 histopathological slides along with their corresponding scanned WSIs. These samples were then divided into three sets: the training set, the testing set, and the validation set, with a distribution ratio of 3:1:1, respectively. To assess the performance of our model, we employed accuracy, sensitivity, specificity, F1 score, ROC curves, and calculated the AUC (area under the curve) values among other evaluation metrics. A two-sided McNemar’s test was performed to compare the differences in accuracy between the optimal deep learning model and the pathologists. These metrics allow us to gauge the accuracy and effectiveness of our model’s predictions in distinguishing *HER2* overexpression. A 95% confidence interval (CI) was calculated using the five-fold cross-validation strategy. *p* < 0.05 was considered statistically significant. These analyses were conducted using Python version 3.10.4.

## 3. Results

### 3.1. Patient Characteristics

After excluding nine cases with poor image quality (one out of nine), inadequate bladder tumor tissue sampling (five out of nine), and incomplete pathological information (three out of nine), we ultimately included 106 patients and their corresponding 106 tissue pathology slides in the Renmin Hospital of Wuhan University (RHWU; Wuhan, Hubei, China) cohort. We incorporated WSIs from 106 bladder cancer patients for training purposes, dividing them into three sets based on a ratio of 3:1:1, namely the training set, test set, and validation set, respectively. Table 2 displays the characteristics of the included patients in the RHWU cohort.

### 3.2. Performance of the Deep Learning Model

We have identified sensitivity, specificity, accuracy, and AUC as the pivotal indicators for assessing the performance of our model. Sensitivity, recognized as the True Positive Rate (TPR), evaluates the model’s proficiency in accurately detecting *HER2*-positive expressions. Conversely, specificity, or the True Negative Rate (TNR), quantifies the model’s effectiveness in identifying *HER2*-negative cases. The accuracy metric offers an overview of the model’s overall success in making correct predictions. The F1 score is the harmonic mean of precision and recall, commonly used to evaluate the performance of models in classification problems. Within our validation set (*N* = 21), the model demonstrated a sensitivity of 0.87 and a specificity of 0.83. However, in the test set (*N* = 21), the sensitivity dropped to 0.56, with the specificity standing at 0.75, indicating areas for improvement and underscoring the importance of further refinement. F1 scores for the validation set and the test set are 86.7% and 77.8%, respectively. (Table 3)

**Figure 3:**
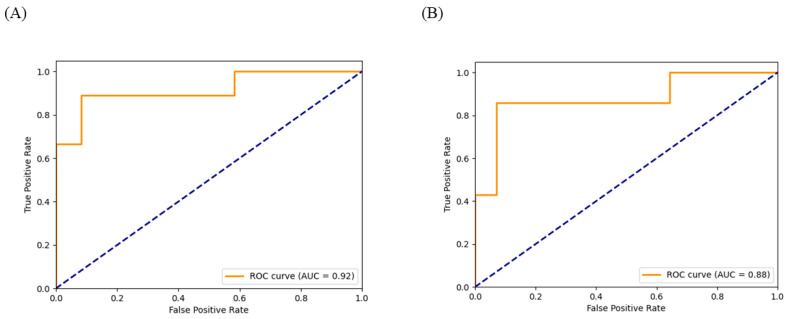
ROC curves of the model in the test set and validation set. (**A**) ROC curve of the model in the validation set. (**B**) ROC curve of the model in the validation set. Orange solid line is the actual ROC curve, indicating the performance of the classification model. It shows the model’s performance at different thresholds by plotting the True Positive Rate (TPR) against the False Positive Rate (FPR). Blue dashed line: This is the diagonal line, representing the ROC curve of a random guess classifier. In this case, the model’s AUC (Area Under the Curve) should be 0.5, indicating that the model has no predictive ability and is equivalent to random guessing.

Out of the total 106 WSIs, the model achieved AUC of 0.92 and accuracy of 0.86 in the validation set; meanwhile, it achieved AUC of 0.88 and accuracy of 0.67 in the test set. (Table 3 and Figure 3).

### 3.3. Human–Machine Competition

To affirm the practicality and efficacy of the deep learning model within actual medical environments, and to bolster the generalizability of our model, we have meticulously implemented an integrative validation protocol. We commenced by adopting the RHWU-Test dataset as our validation set, juxtaposing the model’s diagnostic predictions with the expert evaluations provided by seasoned pathologists. The accuracy metric was selected as the pivotal statistical measure to assess the model’s performance, ensuring a robust and reliable evaluation. Two pathologists were invited to judge the 21 H&E slides in the test set one by one, back-to-back. The results showed that our optimal model outperformed the two senior pathologists, whose accuracy was 0.62 and 0.43, respectively (both *p*-values < 0.05) (Table 4).

### 3.4. Visualizing Deep Learning-Based Predictions

**Figure 4:**
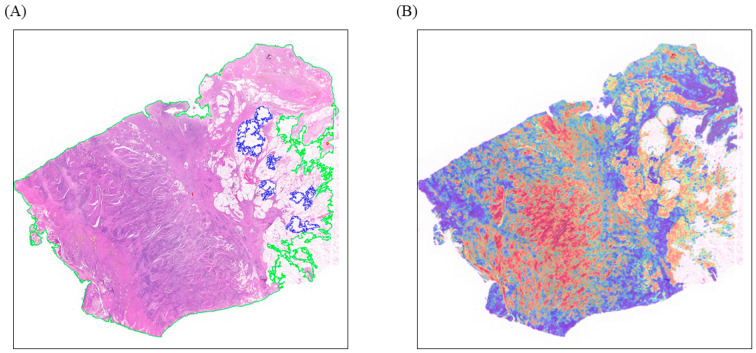
Heatmap visualization.(**A**) Outline of bladder cancer on WSI. (green line is tissue boundary, blue line is holes). (**B**) Heatmap visualization of bladder cancer. The red region indicates areas with higher model activation. These are regions where the model identifies patterns associated with *HER2* overexpression.

Through the CLAM model, we were able to map the *HER2* molecular feature data of bladder cancer onto its pathological image’s spatial locations, thus displaying it in a heatmap. Our findings suggested a strong correlation between the intensely activated red regions in the heatmap and the overexpression of *HER2* (Figure 4). Through heatmap visualization analysis, the attention scores obtained by the original tumor region were higher than those obtained by the surrounding microenvironment tissue region, suggesting that the tumor region might contain more critical predictive information.

## 4. Discussion

With the advent of precision medicine, the prospects for molecular targeted therapies have become increasingly promising [36,37]. Through in-depth research into the mechanisms of bladder cancer, *HER2* has emerged as a significant biomarker for this disease [38]. In this study, our aim was to explore whether the expression of HER2 could be predicted from H&E-stained images using a deep learning-based image analysis model. We collected a dataset of 106 H&E-stained images from patients with bladder cancer, forming the RHWU cohort. Leveraging the CLAM model, we successfully analyzed and predicted *HER2* expression in bladder cancer H&E images with 0.67 mean accuracy. Our research demonstrated that deep learning-based image analysis predictive models can achieve accurate expression predictions of molecular biomarkers.

Both high-throughput sequencing (HTS) and IHC can be used to detect *HER2* overexpression in bladder cancer [39,40]. HTS has high technical requirements, high economic cost, and lack of universality. Although IHC is almost universal, patients still face significant time and economic costs. The deep learning model trained in this study only uses WSIs as the analysis object, which can be easily obtained in the surgical environment and popularized in economically underdeveloped and remote areas. Compared with the former, our model significantly reduces the workload of the pathologist and the pressure of the patient, which is more conducive to the decision of the clinician and the benefit of the patient.

The CLAM model we used showed decent performance in identifying *HER2* in the H&E images of bladder cancer. The patients with bladder cancer included in this study included most TNM stages and almost all pathological stages, which also indicates the universality of our findings. However, the inclusion of cases in each pathological stage is not balanced, which may introduce differences between groups.

In the segmentation process of whole slide images (WSIs), we opt not to initially preprocess the WSIs to eliminate the background. Instead, we segment the WSIs into 256 × 256-pixel patches tailored for convolutional neural networks. We then refine our dataset by employing color thresholding to remove blank patches and proceed with feature extraction using the ResNet-50 model.

ResNet-50 stands out as a sophisticated deep learning architecture with a multitude of compelling benefits: It surmounts the degradation challenge in deep network training via its residual learning framework, thus boosting training efficiency and curbing the vanishing gradient issue. It adeptly learns a spectrum of feature representations—from rudimentary to sophisticated—enhancing the model’s capacity for generalization. The architecture’s modular and scalable design permits customization to meet the demands of tasks with varying complexities by simply adjusting the layer depth. The availability of numerous pre-trained models expedites its application in transfer learning across a spectrum of computer vision scenarios. Moreover, ResNet-50’s computational prowess renders it both swift and pragmatic for real-world deployment. These attributes render ResNet-50 exceptionally versatile, making it well-suited not only for image segmentation but also for a diverse array of visual tasks including object detection and image classification.

During the training of the CLAM model, we utilized the Adam optimizer with an initial learning rate of 1 × 10^−4^ and an L2 weight decay of 1 × 10^−5^ to enhance the model’s generalization and prevent overfitting. The β1 and β2 parameters of the Adam optimizer were set to their default values of 0.9 and 0.999, respectively, which are generally suitable for a variety of tasks. The input feature dimension and hidden layer dimension were set to 1024 and 256, respectively, ensuring that the model has sufficient capacity to capture data characteristics without becoming overly complex. The dropout rate was set to 0.25 to further mitigate overfitting. A smooth top-1 SVM loss function, suitable for classification tasks, was chosen for the loss function. During training, we established a maximum of 200 epochs and implemented an early stopping strategy if the validation loss did not improve for 20 consecutive epochs, to save the best model and avoid excessive training. The comprehensive setting of these hyperparameters aims to achieve an optimal balance between model performance and generalization capability, although further adjustments may be necessary based on the specific dataset in practical applications.

It was found that *HER2* was weakly expressed in bladder cancer from the collected cases of bladder cancer, so the quality of immunohistochemical staining determined the regimen of neoadjuvant chemotherapy or targeted therapy for patients. The use of deep learning techniques to mine more detailed features as objective criteria is potentially beneficial for patients.

The model demonstrated a sensitivity and specificity of 0.87 and 0.83, respectively, on the validation set. However, the sensitivity in the test set stands at 0.56, with a specificity of 0.75, which underscores the need for further enhancement in the model’s capabilities and highlights the potential risk of missed diagnoses. This discrepancy indicates that the model has considerable space for improvement, which we attribute to the use of a single-center sample for training.

Furthermore, Yan et al. introduced a hierarchical deep multiple-instance learning framework that has been applied to predict the *HER2* expression status in bladder cancer tissues and achieved an AUC of 0.91 [30]. Loeffler et al. have illustrated that deep learning possesses the capability to predict the mutation status of the fibroblast growth factor receptor 3 (*FGFR3*) directly from pathological images of bladder cancer, and they identified *FGFR3* mutations with an AUC score of 0.701 [41]. But, compare to the aforementioned study, where the expression status of *HER2* and *FGFR3* was quantified, our research adopts a qualitative approach. Moreover, while their study necessitated the labor-intensive process of annotating bladder cancer tissues, our model eliminates the need for such a procedure, streamlining the diagnostic process.

From a clinical standpoint, our study is poised to alleviate the workload of pathologists, reducing the time and financial burden associated with traditional IHC testing. This streamlined approach has the potential to shorten treatment cycles, leading to improved patient outcomes. Furthermore, during the diagnosis phase, our model can serve as a valuable support, aiding clinicians in making well-informed decisions and enhancing the overall quality of care for bladder cancer patients.

There are still some limitations to this study. Our research data is derived solely from a single clinical center, and the size of our cohort is relatively small. This can impact the accuracy and reproducibility of the results and may limit the applicability of the findings in clinical practice. In addition, there are still some out-of-focus patches mixed in, affecting the performance of the model.

In the future, we envision the inclusion of multi-center studies with large sample sizes in the research focused on *HER2* overexpression in bladder cancer. This approach would enable us to achieve early and accurate diagnosis of *HER2* overexpression in bladder cancer patients, leading to the development of personalized treatment plans and guiding clinical decisions effectively. Moreover, we seek active involvement of pathologists in the research projects, as their expertise can significantly contribute to quality control and overall research improvement. Through the integration of clinical data, radiomics, and gene-related information using multi-instance learning, we anticipate the creation of robust and reliable clinical support models.

## 5. Conclusions

We used deep learning to predict *HER2* status from histopathological images of bladder cancer. Next, we hope to expand the research by conducting a multi-center, large-sample study to reduce overall patient stress and accelerate personalized care.

## Figures and Tables

**Table 1:** Patients included and excluded in the RHWU cohort.

Cohort	RHWU	
Biomarker	HER2	
**Total Patients**	***n***	**%**
Total	115	100
Include From Analysis	106	92.2
Exclude From Analysis	9	7.8
**Include From Analysis**	***n***	**%**
Total Patients	106	100
HER2(+)	70	66
HER2(−)	36	34
Training Set	64	60.4
Test Set	21	19.8
Validation Set	21	19.8
**Exclude From Analysis**	***n***	**%**
Total	9	100
Poor Image Quality	1	1.1
Suboptimal Bladder Tumor Tissue Sampling	5	55.6
Incomplete Pathological Information	3	33.3

**Table 2:** Clinical, biological, and pathological characteristics of bladder cancer patients included in the RHWU cohort.

	RHWU (*N* = 106)
Age (years)	68 (32, 90)
Gender	
Female	15 (14.15%)
Male	91 (85.85%)
pT stage	
pTis	2 (1.90%)
pTa	48 (45.28%)
pT1	23 (21.70%)
pT2	21 (19.81%)
pT3	8 (7.55%)
pT4	4 (3.77%)
pN stage	
pN0	97 (91.50%)
pN1-3	9 (8.50%)
pM stage	
pM0	105 (99.06%)
pM1	1 (0.94%)
pMx	0 (0%)
pTNM stage	
Stage 0a	57 (53.77%)
Stage 0is	2 (1.89%)
Stage I	23 (21.70%)
Stage II	20 (18.87%)
Stage III	3 (2.83%)
Stage IV	1 (0.94%)
Histologic grade	
High grade	61 (57.55%)
Low grade	45 (42.45%)
Missing	0 (0%)
Lymphovascular invasion	
No	31 (29.21%)
Yes	20 (18.87%)
Missing	55 (51.92%)

**Table 3:** Performance of CLAM model to predict HER2 expression.

Cohort	Sensitivity (95%CI)	Specificity (95%CI)	Accuracy (95%CI)	AUC (95%CI)
RHWU-Validation	0.87 (0.63, 0.95)	0.83 (0.53, 0.87)	0.86 (0.74, 0.94)	0.92 (0.86, 0.94)
RHWU-Test	0.56 (0.45, 0.76)	0.75 (0.40, 0.80)	0.67 (0.55, 0.86)	0.88 (0.82, 0.92)

**Table 4:** Performance of HER2 expression predictions by pathologists and optimal model.

	Cohort	Accuracy	*p*-Value
**Optimal Model**	RHWU-Test	0.86	**-**
**Pathologist** A	RHWU-Test	0.62	<0.01
**Pathologist** B	RHWU-Test	0.43	<0.001

## Data Availability

The data presented in this study are available on request from the corresponding authors due to privacy and ethics. The code for CLAM can be accessed at https://github.com/mahmoodlab/CLAM (accessed on 25 August 2023).

## Supplementary Materials

The following supporting information can be downloaded at https://www.mdpi.com/article/10.3390/biomedicines12071583/s1: Figure S1: Poor image quality of bladder cancer; Figure S2: Non-bladder tumor; Figure S3: Suboptimal bladder cancer image; Figure S4: Bladder cancer image.
