# Supplementary figures and images for: Prediction of HER2 Status Based on Deep Learning in H&E-Stained Histopathology Images of Bladder Cancer
Source: Biomedicines. 2024 Jul 17;12(7):1583. doi: 10.3390/biomedicines12071583 (PMC11274957; doi:10.3390/biomedicines12071583)

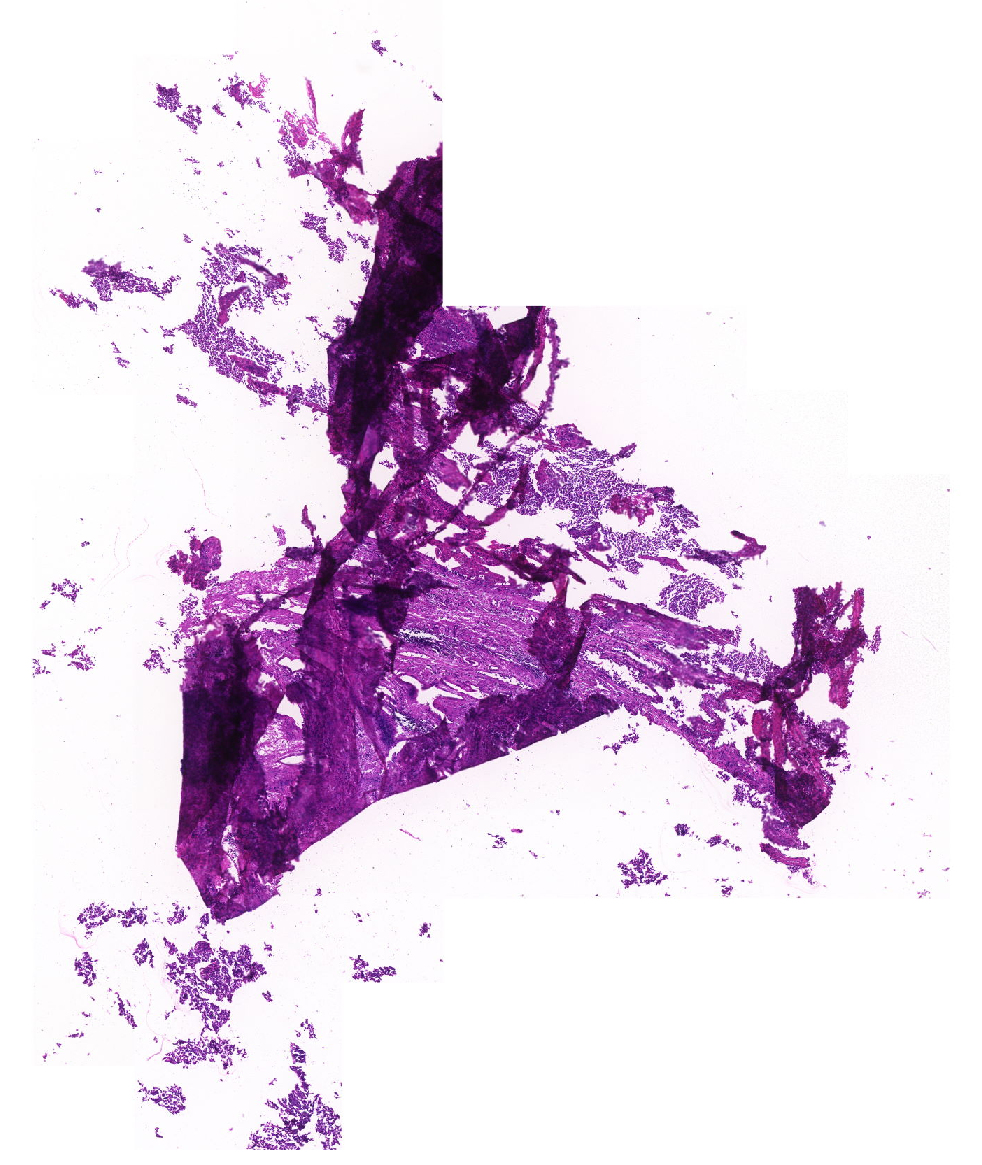

Supplement: Supplementary file 1 [file biomedicines-12-01583-s001.zip › biomedicines-3074994-supplementary/Figure S1.png]

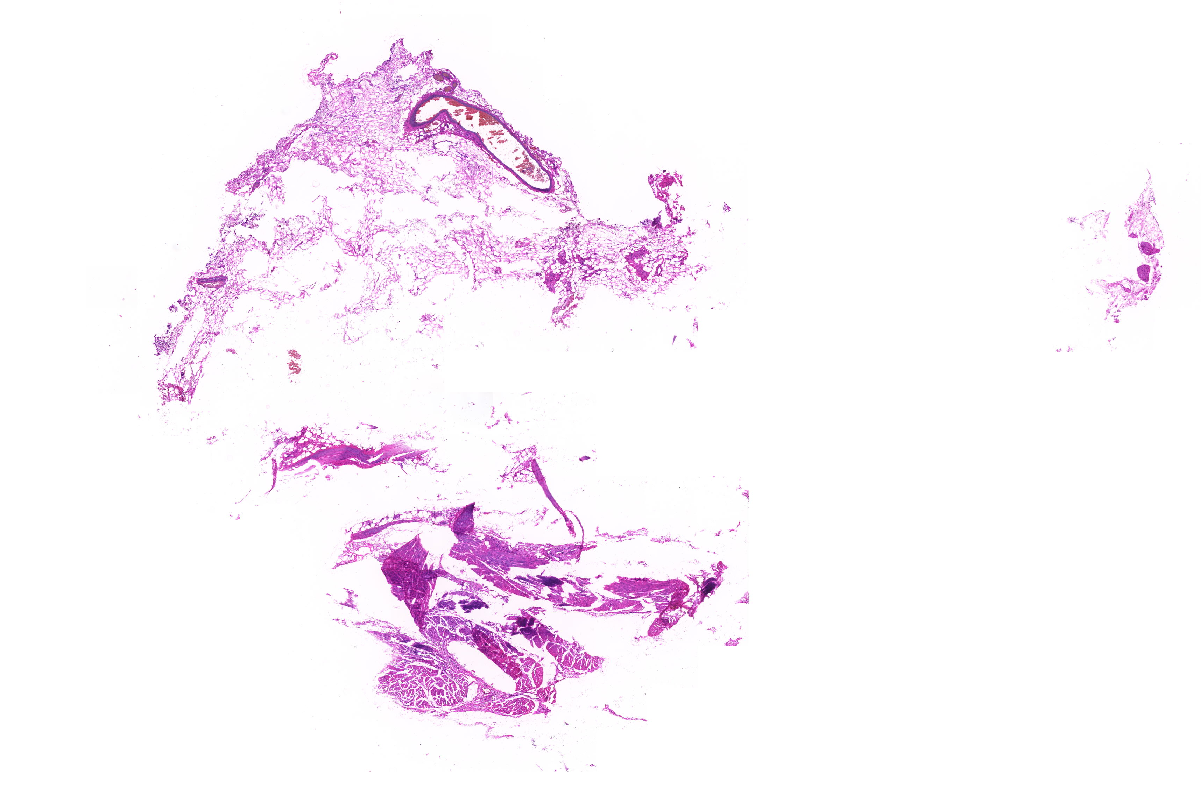

Supplement: Supplementary file 1 [file biomedicines-12-01583-s001.zip › biomedicines-3074994-supplementary/Figure S2.png]

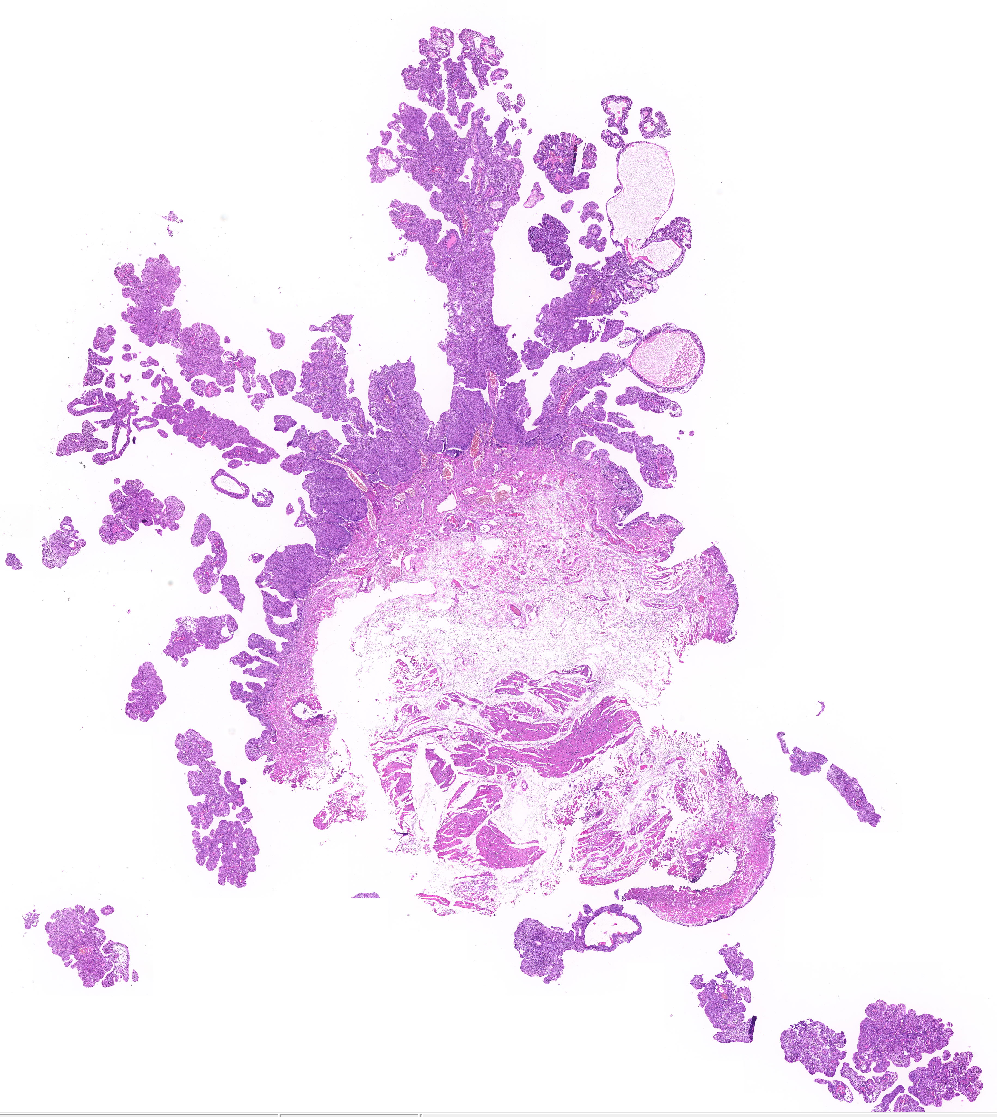

Supplement: Supplementary file 1 [file biomedicines-12-01583-s001.zip › biomedicines-3074994-supplementary/Figure S3.png]

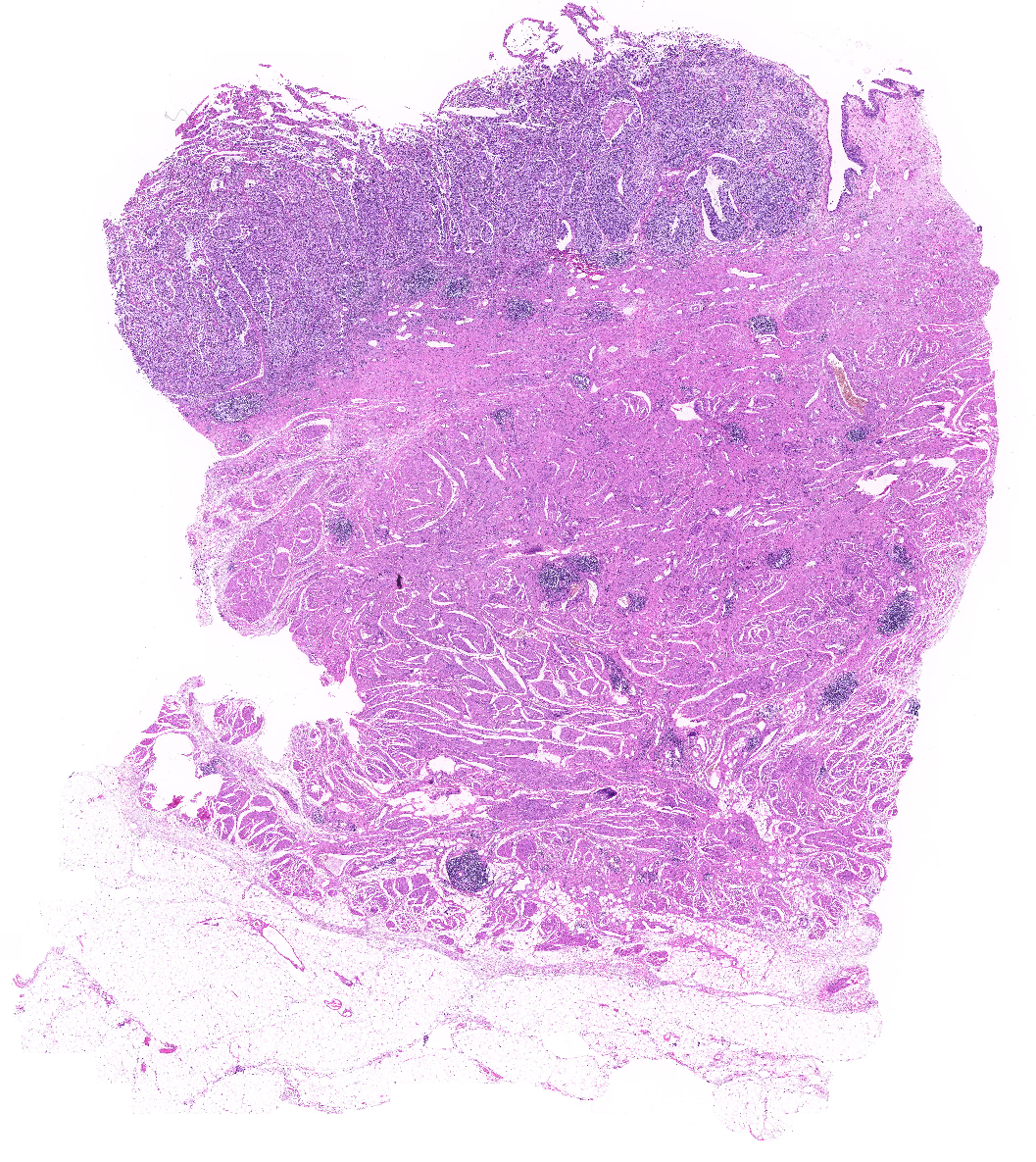

Supplement: Supplementary file 1 [file biomedicines-12-01583-s001.zip › biomedicines-3074994-supplementary/Figure S4.png]
